# Melatonin Relations with Energy Metabolism as Possibly Involved in Fatal Mountain Road Traffic Accidents

**DOI:** 10.3390/ijms21062184

**Published:** 2020-03-22

**Authors:** Claus Behn, Nicole De Gregorio

**Affiliations:** 1 Laboratory of Extreme Environments, Department of Physiology and Biophysics, Institute of Biomedical Sciences, Faculty of Medicine, University of Chile, Santiago 8380453, Chile; nicole.degregorio@gmail.com; 2 Faculty of Medicine, Campus Los Leones, San Sebastián University, Providencia, Santiago 7500000, Chile

**Keywords:** melatonin, dysrhythmia, mountain road death

## Abstract

Previous results evidenced acute exposure to high altitude (HA) weakening the relation between daily melatonin cycle and the respiratory quotient. This review deals with the threat extreme environments pose on body time order, particularly concerning energy metabolism. Working at HA, at poles, or in space challenge our ancestral inborn body timing system. This conflict may also mark many aspects of our current lifestyle, involving shift work, rapid time zone crossing, and even prolonged office work in closed buildings. Misalignments between external and internal rhythms, in the short term, traduce into risk of mental and physical performance shortfalls, mood changes, quarrels, drug and alcohol abuse, failure to accomplish with the mission and, finally, high rates of fatal accidents. Relations of melatonin with energy metabolism being altered under a condition of hypoxia focused our attention on interactions of the indoleamine with redox state, as well as, with autonomic regulations. Individual tolerance/susceptibility to such interactions may hint at adequately dealing with body timing disorders under extreme conditions.

## 1. Introduction

The biosphere oscillates with a near 24-h precision, according to light (and temperature) changes determined by Earth rotation [1,2,3]. Along eons, living beings learned to anticipate recurrent environmental changes and to organize their physiology and behavior correspondingly [4,5,6,7]. A clock network of transcription-translation feedback loops allows mutually antagonistic processes to be temporally segregated. Gluconeogenesis and glycolysis, thus, can respectively prevail during resting and active phases of the day [8]. Individual chronotypes [9] match with the period length of gene expression rhythms in their fibroblasts maintained in culture out of the body [10]. Such rhythms relate to exercise performance being achieved at different daytime [11]. Primordial timekeeping of creatures contrasts, however, with widespread habits of current “24-h society” [2]. People try to keep moving, more or less spinelessly following the monotonous mandate of machines. Perturbations of primordial timekeeping however, associate with mental and physical shortfalls, disease and even shortening of life expectancy [12,13,14]. Metabolic dysrhythmia increases the risk of cardiometabolic diseases [15]. Even perturbations in the timing of food intake may affect metabolic homeostasis, potentially resulting in circadian dysrhythmia between organs [16]. A high-fat meal consumed at the end of the active period of the day, leads to increased adiposity, decreased glucose tolerance, hyperinsulinemia, and decreased cardiac function, as compared to mice fed the same high-fat meal, but during the beginning of the active period [17]. Time-restricted feeding, on the other hand, shifts the peak of respiratory quotient oscillations and increases their amplitude as compared to ad libitum-fed mice [18], and tends to prevent metabolic disease [19]. Circadian dysrhythmia implicates decrements of performance [20,21,22,23] that, in turn, represent a main cause of accidents. The latter becomes especially relevant in the case of human work realized in unusual environments. Large scale mining in Chile, mostly realized above 3000 m about sea level (m asl), thus, reached in 2013, an annual incidence of 25 fatal accidents/100,000 workers [24]. This number quintuplicates the mean incidence of deaths at work in Chile, the latter number already doubling the corresponding average in OECD countries. Present day work-related time schedules often collide also in habitual environments with endogenous rhythmicity of body functions. We have still much to learn concerning how to balance natural body timekeeping with modern lifestyle and ambitions. An increased interest in rhythms is, therefore, justly noticed in health care and medicine [25]. 

## 2. Rhythm Generation

Life implicates and depends on rhythms. Energy flowing through living beings generates linear and/or cyclic deviations from thermodynamic equilibrium [26] “Nondecaying, repetitive phenomena”, or cycles, determine the chemical organization of living matter [27]. Cycles also occur in simple organic chemical processes like the Belousov-Zhabotinskii reaction [28]. Oscillatory reactions often involve positive free enthalpy (Durup, 1979 cited by [29]). An oscillating signal requiring less energy implicates a gain in precision of control [30]. Biological control based on periodical events, thus, becomes energetically more advantageous and more efficient than that relying on steady-state reactions [30]. Phased with the environment at more or less constant period length, endogenous oscillators influence the genome [5] and by means of the latter, also affect functions like cell growth, protein synthesis, stress responses, and intermediary metabolism [31]. Timekeeping of energy metabolism and related body functions is mostly under the control of transcriptionally based, cell autonomous mechanisms in step with post-translational processes. Interlocked by transcriptional feedback loops, gene products like CLOCK and BMAL1 drive biological clocks positively, while others like CRY1-2, PER1-3 and REV-ERBα do it negatively [5,7,31,32]. Individual phase differences in PER3 expression, thus, correlate with timing of sleep during a constant routine [33]. The mechanisms by which the multiple feedback loops are integrated remain largely to be specified. Uncertain are also the post-translational mechanisms possibly involved in clock regulation [14,34,35,36]. Clock components (such as BMAL1 and CLOCK) may, moreover, additionally affect gene expression and metabolic processes, apparently not related to their proper timing function [15,37]. The mechanism by which clock protein stability connects with circadian period length is not yet exactly known [38]. Clinical consequences of period shortening in circadian cycles are evidenced in sleeping sickness caused by *Trypanosoma brucei* [39]. Noticeably, however, cycling also occurs in the absence of a genome, as shown by intracellular K^+^ levels in human red cells [40]. However rhythms are caused, they allow the necessary time keeping in living beings, or as having stated by Joseph Bass (2017): *As energy metabolism being in constant flux, there would be time in biochemical processes, as there is in a central train station* [41].

## 3. Rhythms, an Information System

Periodic processes tend to function in coupled entities [42,43,44]. The phase response of a single oscillator, thus, can sustain timing behavior at the multi-oscillator level [45]. From daily repetition of steady phase relationships among multiple clocks, a temporal order of the whole organism can arise. Interrelated clocks seem to constitute together a complex information handling system, keeping the organism as a whole in step with the challenges imposed by a periodically changing environment [30,46,47,48,49]. Thus, also the central circadian system represents an ensemble of oscillators crossing different brain regions [31]. Rhythms of body temperature, thus, also relate to locomotor activity [50,51], both together determining exercise performance [52,53]. Activity of brown adipose tissue seems, moreover, be involved in this context [54,55,56]. Oscillations of body core temperature, noticeably, coinciding with the daily sleep-wake cycle, make a phase advance already in human midlife [46,57,58]. Daily repeating phenomena, thus, coordinate widespread functions like temperature control [59,60,61], cardiovascular performance [62], autonomic [63], and endocrine regulations [49,64,65], as well as, behavior [52,53,66,67]. Biological oscillators, thus, effectively contribute to sustain homeostasis [30,68] and related functions like preconditioning (hormesis) and learning [69,70,71,72,73,74]. Even everyday coupling of rhythms between subjects has a neurophysiological substrate [75]. 

## 4. In the Beginning There Was Light

Environmental rhythms are coupled in homeothermic vertebrates with endogenous oscillators by a light sensitive neurohumoral network that includes retina, retinohypothalamic tract, nucleus suprachiasmaticus, pars reticularis, and the epiphyseal hormone melatonin [76,77,78,79,80,81,82]. Melatonin, an ubiquitous indoleamine, crucially influences the life of bacteria [83,84], unicellular algae [85], higher plants [86,87,88,89], and animals. The synchronizing role of melatonin in higher animals extends from the central nervous system up to peripheral oscillators in the cardiovascular system, skin, liver, adrenals, and various primate fetal tissues. Beside its crucial role as a circadian rhythm regulator, melatonin functions as a free radical scavenger and antioxidant agent [84], as well as, a neuroprotective [90], anti-inflammatory [91,92], and immunoregulating molecule [91], and even an oncostatic factor [84,93,94]. Melatonin, moreover, enhances carotid body chemoreceptor sensitivity [95] and lowers body temperature [77,96,97]. A normal daily schedule, may strengthen, on the other hand, circadian rhythmicity even under conditions of compromised light/dark cycles [98]. Melatonin not only responds to circadian light/dark cycles and seasonal differences of light’s impact on plants and animals [14]. The indoleamine appear also be regulated by food intake [31], involving factors like ghrelin [99] and orexin A [100]. Additional cues for clock functions include learned patterns like physical exercise routines [101]. Noticeably, cycles in tissue oxygenation appear to synchronize cellular clocks depending on hypoxia-induced factor 1-alpha (HIF-1α) [102]. 

Changes in environmental light [103], particularly concerning day length—as well as light intensity [104] and wavelength prevalence [105]—affect circadian rhythmicity. Light/dark changes affect almost all body functions [80,106,107,108]. Light in the night disrupts endocrine rhythms of living beings accustomed to being active during the day [109,110]. Light emerging from human affairs distorts bird reproductive behavior and mating patterns [111]. Melanopsin, an ancient bistable photopigment contained in “intrinsically photosensitive retinal ganglion cells” (RGCs), initiates the signal transduction by which light drives circadian rhythms [112]. Peak activation of intrinsically photosensitive RGCs occurs by light in the range of 460–480 nm, coinciding with the maximal absorption spectrum of melanopsin. Blue-light (480 +/− 20 nm) triggers the melanopsin photoreceptor system related to circadian rhythm control [113]. RGCs convey light induced signals along the retinohypothalamic tract to a master clock in the suprachiasmatic nuclei (SCN) of the basal hypothalamus for ulterior control of peripheral tissues. The master clock in SCN sensitizes peripheral molecular clocks by neurohumoral means to the light/dark cycle, thereby integrating circadian rhythmicity at the whole-body level [112]. The principal photic resetting cue to the SCN is in the 470 to 480 nm range, the range of wavelengths responsible for maximal RGC activity. Prior exposure to long-wavelength light may enhance SCN responses to 480 nm light [114]. 

However, it is in the epiphysis, or pineal gland where—according to Descartes—the soul interacts with the body via “vital spirits” [115,116]. The pineal gland provides melatonin (N-acetyl-5-methoxytryptamine) by N-acetyltransferase mediated cleavage of serotonin, the latter derived from the plant amino acid tryptophan [117]. Coincident with the absorption spectrum of melanopsin, blue light suppresses nocturnal melatonin secretion with maximal efficiency in the 446 to 477 nm range [118]. Filtration of a 10-nm bandwidth of light between 470 and 480 nm prevents light-mediated melatonin suppression. Blocking all wavelengths less than 530 nm, on the other hand, enhances melatonin secretion [119,120]. Melatonin cycles are altered, sleep is disrupted, and symptom prevalence is elevated in night workers [121]. Risks for two or more symptoms were 3.5 to 8 times greater among workers with sleep:work ratios < or =1 than those with ratios >1 [21]. Controlled reduction of short wavelengths in polychromatic light may prevent negative impacts on cardiac physiology without affecting cognitive performance and alertness in night-shift workers [122]. It must be considered, however, that the photic stimuli applied in this work included beside the short wavelength restriction, a very high irradiance level. This circumstance may relativize the above effect of short wavelength restriction. Interventions on melatonin secretion are difficult to apply in rotating shifts [123] and may have adverse effects on health [124].

**Figure 2:**
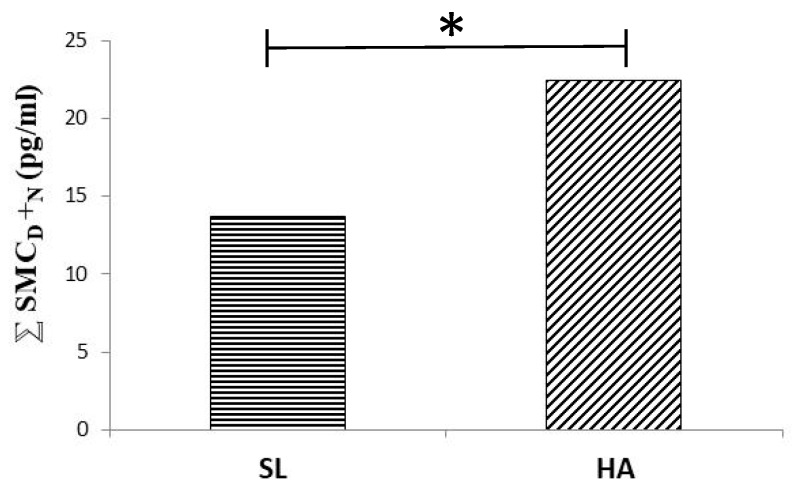
The sum of total (night and day) SMC at SL (left) and HA (right). Asterisk represents statistical difference (*p* < 0.05).

A high altitude (HA)-related defect in blue axis vision and discrimination may affect non-visual brain responses to light, including defects in circadian rhythm control. Amplitude of circadian melatonin rhythm diminished and its relationship with respiratory quotient (RQ) weakened at a moderate altitude (Figure 1 of [125], with permission to be asked). Vision defects may be implied in melatonin rhythm alteration at HA. Exposure to HA alters color vision [126,127]. While protan (red) and deutan (green) axis discrimination seems still to be normal at 5400 m, tritan (blue) axis vision and discrimination are, on the contrary, reduced at HA [127,128,129]. Intense physical exercise seems to have a similar effect [130]. Melatonin secretion [131] and urinary 6-hydroxy-melatonin sulfate excretion [132] (derived from data contained in [125]), correspondingly, increase on acute exposure to HA. Summing up midday and midnight values of salivary concentration of melatonin renders a higher figure at 3700 m ASL than at sea level (Figure 2). The mechanism by which HA affects blue-light vision and, thereby, potentially also circadian rhythm control, remains to be elucidated. RGC have, indeed, been shown to be extremely hypoxia-sensitive [133]. Beta-adrenoceptors appear to trigger melatonin synthesis [134]. Hypoxia-related sympathetic stimulation may thus be also involved in diurnal increases of melatonin at high altitude. Overabundance of short-wavelength-enhanced light at HA [135] caused by Raleigh scattering [136] may be expected to induce protective mechanisms. Short-term neuronal plasticity in the retinohypothalamic tract synapses of suprachiasmatic nuclei [137], thus, may lead to synaptic depression [138]. Protection of SNC neurons by synaptic depression has also been advocated to favor clock adjustment in response to slow changes in the circadian light-dark cycle. Lack of light-induced melatonin suppression may be expected to cause somnolence at work and insomnia during the rest period, not only at HA, but in all working places without appropriate illumination. It should be noticed, however, that complex conditions are present in night-shift work, the real-life situation involving not only lighting. Light-induced melatonin suppression, however, clearly being impaired at HA, adds to an impressively large list of factors potentially inducing dysrhythmia at HA, including hypoxia, synaptic depression, sleep/wake cycle alterations, changes in food intake schedule, and last, but in our time, not least, the inescapable shift work. 

## 5. The Redox System, an Axis

Reactive oxygen species (ROS), lipid peroxidation, and nuclear factor kappa B (NF-κB) protein expression levels—as well as transvascular leakage—increase in the brains of rats exposed to a simulated altitude of 25,000 ft [139]. Mainly in response to stress, and at first glance, unrelated to circadian oscillation, melatonin is synthesized in the mitochondrial matrix of mice brains [140]. Once released into cytoplasm, melatonin activates a mitochondrial MT1 signal-transduction pathway. The MT1 pathway inhibits stress-mediated cytochrome C release and caspase activation, thereby protecting against neurodegeneration [90]. Melatonin, moreover, promotes the expression of sirtuin I (a histone deacetylase), thereby enhancing the amplitude of circadian oscillations and promoting survival [141]. Clock mutation in mice diminishes circadian pacemaker amplitude but leads resetting stimuli to be more efficient [142]. Receptors of melatonin in the SCN appear to require G-protein-coupled [90], inwardly rectifying potassium (GIRK) channels, thus participating in a widely distributed physiological neural communication system [143]. The potential usefulness of melatonin receptor-agonists to address sleep problems [144] may be seen in this context (but see also [145]). 

Tissue melatonin content increases in response to stressful conditions in plants [146], as well as in animals [131,132]. Beyond its role in supporting circadian rhythmicity, melatonin protects via antioxidant [84,147,148,149], anti-inflammatory [91,92], and oncostatic effects [94]. Melatonin limits ROS formation [150] and reduces photosynthesis, a known source of ROS [85]. Melatonin counteracts oxidative [151] and nitrosative [152] stress. The indoleamine scavenges ROS and nitrogen (RNS) reactive species [153]. Enhanced photo-consumption of melatonin by free radicals contributes to decrease the indoleamine on exposure to daylight in *Symbiodinium*, a dinoflagellate, but may have varied before rising irreversibly some 2.4 billion years ago during the Great Oxidation Event (GOE), by enhanced free radical production in relation with daily light/dark changes [85]. Melatonin also enhances antioxidant enzyme activities [154] and regenerates endogenous antioxidants like glutathione [155]. Relations of melatonin with oxygen may be traced back to emergence of the latter gas in the Earth’s atmosphere [83]. Atmospheric oxygen raised irreversibly during the GOE some 2.4 billion years ago [156]. Circadian melatonin oscillations in *Symbiodinium* are thought, indeed, not to be caused by endogenous circadian control, but rather by variations in photo-consumption of atmospheric oxygen [85]. 

Oxygen shortage occurring at HA may serve as a model for effects of cardiovascular and respiratory failures, strenuous physical exercise, pregnancy, ageing, inflammation, and terminal cancer. Hypobaric hypoxia of HA implicates alterations of energy metabolism [157], including oxidative stress [158]. ROS mediate, at least in part, tissue damage related to hypoxia and subsequent reoxygenation [159]. Repair of hypoxia related tissue damage requires energy. Lack of oxygen decreases the production of ATP but, for repair, concomitantly also increases energy demand. Reducing energy requirements in the presence of oxygen shortage, thus, can prevent hypoxia from occurring. The potential effects of hypoxia on mental and physical work capacity thus may be mitigated by increasing the availability of antioxidants. Lack of oxygen is also known to blunt the amplitude of circadian oscillations of oxygen consumption [160,161], with potential consequences for almost all body functions [60,161]. In adult rats, hypoxia (10.5% O_2_ for three days) reduced the amplitude of daily basal temperature oscillations by 55% in adult female rats, and 22% in adult male rats [161]. Hypoxia-related redox alterations may be suspected to be involved in HA-related dysrhythmia. H_2_O_2_ cycles have recently been found to be essential for clock function, as related to energy metabolism [162] see also [163,164,165,166,167]. Shift work, disordered food intake, and melatonin-related neurohumoral dysrhythmia may conflagrate in decrements of mental and physical performance occurring on exposure to HA. Normalizing redox cycles at HA may improve dysrhythmia under the latter condition.

Hypoxia, the lack of oxygen, as related to ATP requirements [168] affects biological clocks [169,170,171,172]. Hypoxia alters circadian rhythms of *Drosophila* [173], rats [174,175], and humans [176,177]. Circadian patterns of gene expression [178] and mitotic activity [179] are also affected by hypoxia. Adult rats having been exposed to hypoxia during their gestation, show diminished activity levels, phase-advanced activity rhythms, and delayed adjustment to light–dark perturbation [180]. Relative amplitude of daily oscillations is nearly constant among species [181,182], but appears to be drastically decreased by hypoxia. Hypoxia affects circadian oscillations of body temperature and metabolic rate [171]. As the latter variables influence almost all body functions [60], hypoxia may perturb all temperature dependent functions [172]. Acting as a hypothermic agent [77,96,97], melatonin may also protect.

Generating a surplus of electrons, hypoxia promotes free radical reactions [183]. ROS affect the structure of lipids [159,184], proteins [185,186], and nucleic acids [187]. Malondialdehyde (MDA), a lipid peroxidation product, relates in exhaled breath condensate (EBC) with severity of acute mountain sickness (AMS) in climbers exposed to an altitude of 5000 m asl [184]. MDA concentration in EBC also rises in response to acute cycloergometric exercise realized at 2200 m asl, but not at 670 m asl [184]. Lipid peroxidation correspondingly increases in response to physical exercise performed while the inspired fraction of oxygen is lowered to 0.16 [188]. Hypoxia also increases eicosanoid plasma concentration [189]. Extending to microsomal membranes, lipid peroxidation may further enhance oxidative stress. Melatonin appears to modulate redox status in pulmonary vessels along gestation [190]. 

Hypoxia leads to a somewhat stereotypic sequence of events in rather enclosed organs like brain, lungs, and perhaps also testis. Lack of oxygen primarily affects active transport implicating cellular salt and water accumulation. Brain white matter volume correspondingly increases in response to a subject’s exposure for 22 h to HA [191] but see also [192]. An increase of cell volume increases tissue pressure in organs unable to expand. The increase of tissue pressure reduces vascular transmural pressure. The capillary bed thus diminished accentuates the lack of oxygen. Ensuing cell damage releases alarmins, activating NF-κB, an ubiquitous, pleiotropic, and pro-inflammatory transcription factor. NF-κB is activated by hypoxia [193,194,195], even in Hela cell cultures [196,197] and in vessels [198]. NF-κB activation by hypoxia also leads to activation of HIF-1α. Concomitant activation of HIF-1α triggers the expression of hundreds of genes. Noticeably, melatonin inhibits HIF-1α [151]. Melatonin protects gastroduodenal mucosae [199]. HIF-1α-mediated lesions of the gastrointestinal tract have been observed on acute exposure to HA [200]. By inhibiting the angiogenic effect of HIF-1α [201], melatonin may also be included in the arsenal of antineoplastic agents. Varicocele (VC), a dilatation of scrotal portion of pampiniform plexus and the internal spermatic venous system [202], occurs among Chilean miners with an incidence directly related to geographical altitude of their usual working site. In a cohort of 465 miners working at different levels A (<2400 m; *n* =167), B (3000–3900 m; *n* = 86) and C (>3900 m; *n* = 243) the incidence of Grade 1 VC (not visible, but palpable), was respectively 4.4%, 9.5% and 36.9% (Marchetti, N (Faculty of Medicine, University of Chile, Santiago, Chile). Personal communication. 2020). Experimental VC upregulates HIF-1α/BNIP3/Beclin1 autophagy-signaling pathway in testicular tissue, revealing a condition potentially promoting male infertility [203]. Repair of experimental VC, on the other hand, reverts the presence of HIF-1α in rat testis [204].

## 6. No Swing, No Pleasure, No Health

Humans. chronically ignoring their intrinsic rhythmicity. Feel sick [12], report fatigue and somnolence [205], suffer from mood disorders [108,206,207,208], and tend towards drug abuse [209,210]. Psychiatric morbidity appears to be elevated in extreme human chronotypes, even under normal working conditions [211,212,213]. Shift work, thus, affects health [108,214,215], decreases mental and physical performance, and may even shorten life expectancy [62,216,217,218,219,220,221]. Hormonal imbalances associated with shift work include alterations of leptin and insulin levels [221,222,223]. Lack of leptin decreases fatty acid metabolism in nonadipose tissues, including the myocard [224]. Energy homeostasis, thus disrupted, leads to oxidative stress and telomere attrition [225], and derives into pathologies like metabolic syndrome [219], obesity [219,223,226], and type 2 diabetes mellitus [227,228]. Both long- (e.g., adiposity) and short-term (e.g., glucose/lipid tolerance) metabolic homeostasis are altered by circadian clock disruption [15]. Feeding during sleep phase increases adiposity in wild-type mice [17]. Long-term costs of night-shift work [121], moreover, involve risks of developing cardiovascular disorders [62] including arterial hypertension [221,229,230]. Accelerated aging [231], cancer promotion [232,233,234,235], breast [236,237] and colorectal cancer [238] profile in this respect. Mechanisms involved in circadian rhythm generation and control—as well as environmental factors potentially affecting them—thus must be considered for prevention and intervention of shift work related risks.

Feeding dysrhythmia, hyperphagia, obesity, and evidence of metabolic syndrome occur in homozygous clock-mutant mice [223]. However, longstanding misalignments between endogenous rhythms and exogenous cues [239,240] can affect health and survival of living beings, from plants [43,86,87,88,240] up to higher animals [231,241]. Uncoupling of endogenous biological rhythms from light changes determined by rotations of the earth can affect almost all body functions [80,106,107,108,242]. Heart rate and locomotor activity of rodents—usually resting during the day—diminish in response to light applied in the night [243]. Light in the night disrupts endocrine rhythms [109,110,222]. Altering bird reproductive behavior and mating patterns by light emerging from human affairs distorts previously reliable quality-indicator traits [111].

## 7. Death in the Mountain

**Figure 3:**
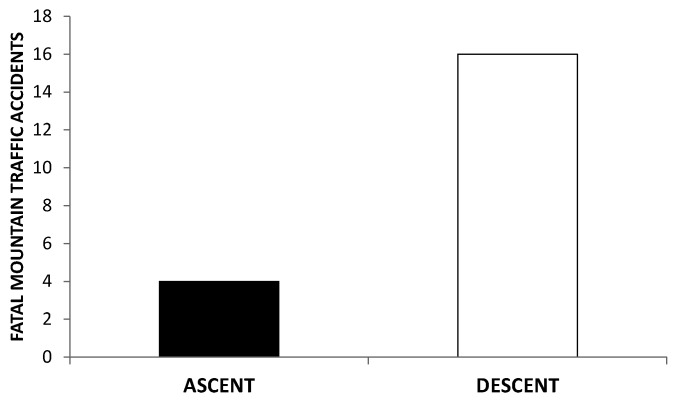
Fatal road traffic accidents in Chilean mining (*n* = 18) and Atacama Large Millimeter Array (ALMA) Observatory (*n* = 2) between the years 2005 and 2017. Column on the left (*n* = 4) and the right (*n* = 16), respectively, indicate the number of cases that occurred during ascent and descent (De Gregorio, N (Faculty of Medicine, University of Chile. Santiago, Chile). Unpublished results. 2020).

**Figure 4:**
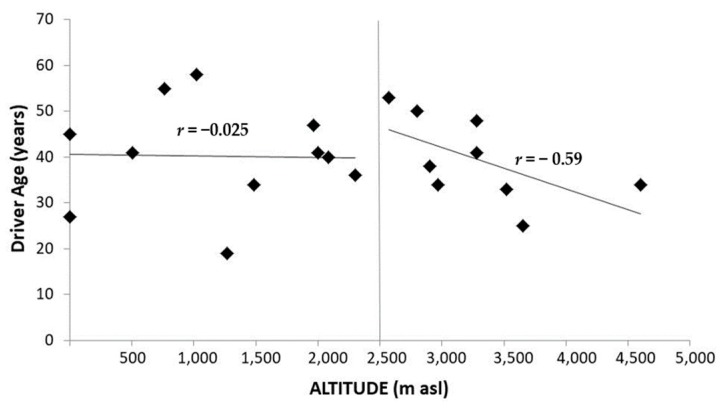
Fatal road traffic accidents in Chilean mining (*n* = 18) and ALMA Observatory (*n* = 2) between the years 2005 and 2017, comparing the age of the deceased driver in terms of the altitude at which the accident occurred. The younger the subjects are, they appear to be more susceptible to suffer fatal accidents at altitudes from 2500 m ACL upward (*r* = −0.59) (De Gregorio, N (Faculty of Medicine, University of Chile. Santiago, Chile). Unpublished results. 2020). Notice that subjects younger and older than 40 years appear rather evenly distributed on the right-hand side of the graph.

Death menaces at HA, particularly at extreme altitudes. Shift work and related sleep deprivation [244,245,246], food intake disorders, permanent alertness, as well as hypoxia, combine to involve body timekeeping disorders that substantially contribute to endanger any human work on the mountain [247,248,249,250]. Moreover, healthcare facilities along mountain roads are often scarce, implicating additional difficulties for rescue teams to arrive in time. Mining and astronomy are mainly realized at HA (above 3000 m asl) in Andean countries like Chile. Dangers due to dysrhythmia in the mountain concern particularly road traffic. At least three conditions are to be considered in road traffic at HA: velocity of ascent, the altitude reached, and time spent at HA [249]. Difficulties are typically noticed by drivers at 2500 m asl [247], or 3000 m asl upward [250]. Physiological conditions for commuting at HA should differ substantially between ascent and descent. Among deaths registered above 8000 m asl on Mt Everest over an 86-year period, only 10% occurred during ascent. On the other hand, during descent from the summit, the death rate of climbers nearly sextuples that of Sherpas [251,252]. Information provided by Atacama Large Millimeter Array Observatory (ALMA) and National Geology and Mining Service of the Chilean Government, (SERNAGEOMIN) allowed us to derive the incidence of fatal road traffic accidents at HA sites in Chile. Retrospectively it was possible to distinguish whether each accident occurred on ascent or descent as seen in Figure 3 (De Gregorio, N (Faculty of Medicine, University of Chile, Santiago, Chile). Unpublished results. 2020). Noticeably, not only the incidence of fatal road traffic accidents (Figure 3), but also that of cardiac arrhythmias [248] are higher on descent than on ascent. The incidence of cardiac arrhythmias on descent is, moreover, higher in drivers younger than 40 years than in older ones [248]. Figure 4, correspondingly, shows an inverse relationship between fatal road traffic accidents and age of the driver above 2500 m asl (De Gregorio, N (Faculty of Medicine, University of Chile, Santiago, Chile). Unpublished results. 2020) Dysrhythmia of melatonin at HA [125] may be considered in this respect, particularly concerning some not yet fully-explored relations of indoleamine with autonomic nervous system (ANS). Risk-determining hemodynamic features along ascent and descent, respectively, depend on dominance of the sympathetic and the parasympathetic branches of ANS. Hypoxia, the prevalent challenge on ascent, implicates sympathetic stimulation [253], the latter presumably associated with enhanced melatonin secretion [134]. An overabundance of melatonin—provoked by sympathetic dominance during ascent—will most probably impact potentiating vagal activity; the latter being enhanced by subsequent reoxygenation during descent. Oxygen availability, being restored along descent, leads, indeed, to a rebound of parasympathetic tonus [254]. Vagotonusinversely relates to age [255,256] and tends to be augmented by melatonin [257,258,259]. Enhanced vagal activity can lead to accidents by provoking failures in consciousness. Dangerous absences while driving may particularly be expected to occur in relation with combined vagal effects on heart rhythm [248], vascular tone [260], and blood glucose levels [261]. Bradycardia induced by enhanced vagal activity implies augmented vulnerability to generation of extrasystoles emerging from ectopic foci. Melatonin further enhances the interval between heart beats, at least in supine humans [262]. Depending on its receptors (MT1 and MT2), melatonin exhibits concentration related cardiovascular effects [263,264,265] that, not being less important for circulatory and metabolic pathology [266] are beyond the scope of the present review. The incidence of arrhythmic events in truck drivers is higher during descent than ascent [248]. Any arrhythmic event—and the related cerebral hypoperfusion—may trigger absences leading to accidents. Moreover, vagal hyperactivity may involve venous dilatation, an effect also potentiated by melatonin, promoting blood pooling in the legs. A compensatory postural tachycardia is, on the contrary, reduced by melatonin [267]. Reflex sympathetic responses to orthostatic stress are diminished by melatonin [257]. Vagal tendency to promote orthostatic collapse, thus, possibly being potentiated by melatonin, may successively curtail venous return, diastolic filling of the heart, cardiac output and, hence, also cerebral perfusion. Vagal hyperactivity may also enhance insulin secretion. Cognitive shortfalls promoting accidents on descent may thus, also result from hypoglycemia. Triggering tidal secretion of melatonin, by transcutaneous vagus nerve stimulation has been shown to lower blood glucose levels in Zucker fatty rats [268].

## 8. Conclusions

**Figure 5:**
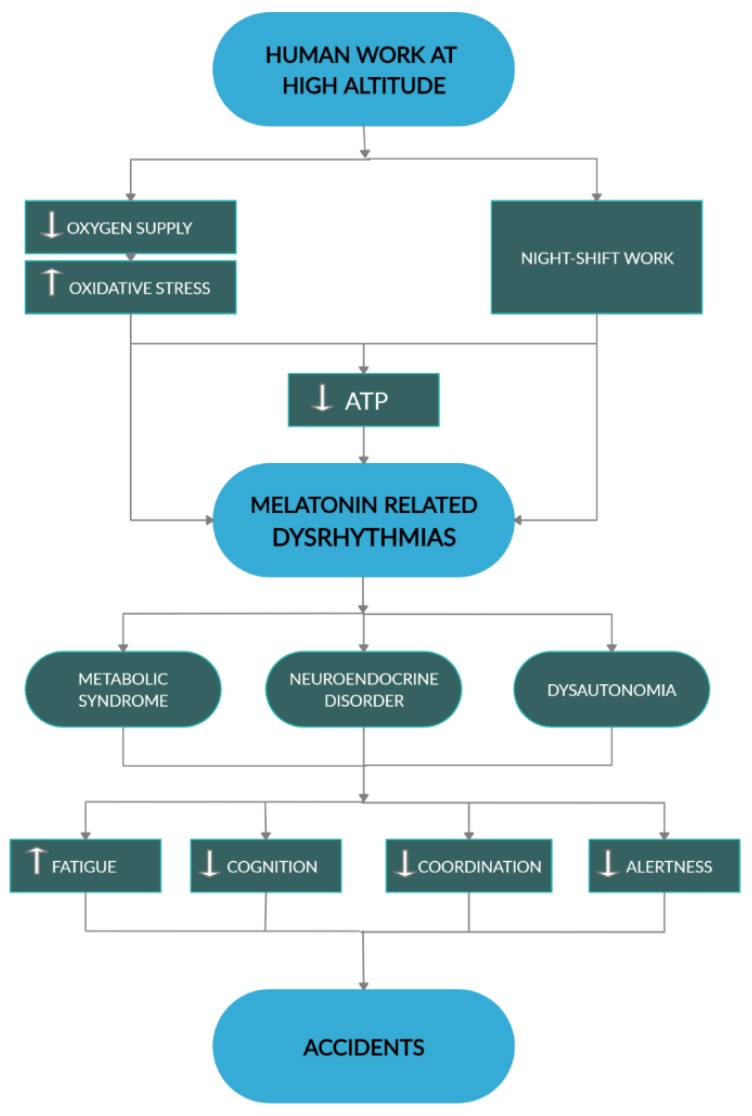
This diagram summarizes dysrhythmia centered mechanisms by which human work at HA may promote accidents. White arrows, respectively indicate increase and decrease. Black arrowheads indicate direction of processes.

All in all, defective melatonin interactions with energy metabolism, particularly affecting redox status and autonomic regulations, may substantially contribute to current death tolls for working in extreme environments (Figure 5). 

A more detailed knowledge on the mechanisms determining individual susceptibility under such conditions will give, in this respect, some outlook into a brighter future.

## Figures and Tables

**Figure 1:**
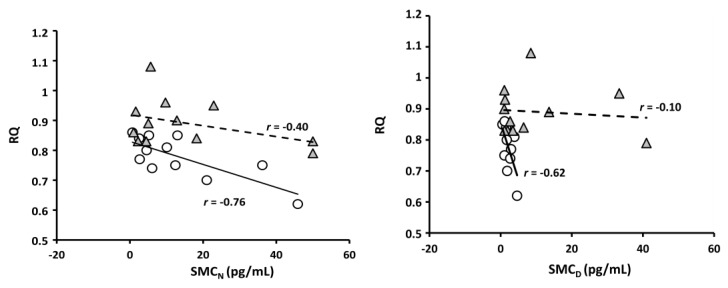
Salivary melatonin concentration (SMC) as related to respiratory quotient (RQ) (permission to be asked, Tapia et al., 2018) in *n* = 12. At the left, the relation between RQ and SMC at 12:00 PM (SMC_D_) at sea level (white circles) and at high altitude (HA) (grey triangles). On the right side, the same relation at 12:00 AM (SMC_N_), also at sea level and HA.

## References

[1] Bünning E. (1936). Die endogene Tagesrhythmik als Grundlage der Photoperiodischen Reaktion. Ber. Dtsch. Bot. Ges..

[2] Moore-Ede M.C. (1986). Physiology of the circadian timing system: Predictive versus reactive homeostasis. Am. J. Physiol..

[3] Aschoff J. (1984). Circadian timing. Ann. N. Y. Acad. Sci..

[4] Pittendrigh C. (1993). Temporal Organization: Reflections of a Darwinian Clock-Watcher. Annu. Rev. Physiol..

[5] Sassone-Corsi P. (1998). Molecular clocks: Mastering time by gene regulation. Nature.

[6] Wilczek A.M., Burghardt L.T., Cobb A.R., Cooper M.D., Welch S.M., Schmitt J. (2010). Genetic and physiological bases for phenological responses to current and predicted climates. Philos. Trans. R. Soc. B Biol. Sci..

[7] Rijo-Ferreira F., Takahashi J.S. (2019). Genomics of circadian rhythms in health and disease. Genome Med..

[8] Kohsaka A., Bass J. (2007). A sense of time: How molecular clocks organize metabolism. Trends Endocrinol. Metab..

[9] Chua E.C.P., Shui G., Lee I.T.G., Lau P., Tan L.C., Yeo S.C., Lam B.D., Bulchand S., Summers S.A., Puvanendran K. (2013). Extensive diversity in circadian regulation of plasma lipids and evidence for different circadian metabolic phenotypes in humans. Proc. Natl. Acad. Sci. USA.

[10] Brown S.A., Kunz D., Dumas A., Westermark P.O., Vanselow K., Tilmann-Wahnschaffe A., Herzel H., Kramer A. (2008). Molecular insights into human daily behavior. Proc. Natl. Acad. Sci. USA.

[11] Facer-Childs E., Brandstaetter R. (2015). The impact of circadian phenotype and time since awakening on diurnal performance in athletes. Curr. Biol..

[12] Rea M.S., Bierman A., Figueiro M.G., Bullough J.D. (2008). A new approach to understanding the impact of circadian disruption on human health. J. Circadian Rhythm..

[13] Kervezee L., Kosmadopoulos A., Boivin D.B. (2020). Metabolic and cardiovascular consequences of shift work: The role of circadian disruption and sleep disturbances. Eur. J. Neurosci..

[14] Gorman M.R. (2020). Temporal organization of pineal melatonin signaling in mammals. Mol. Cell. Endocrinol..

[15] McGinnis G.R., Young M.E. (2016). Circadian regulation of metabolic homeostasis: Causes and consequences. Nat. Sci. Sleep.

[16] Bray M.S., Ratcliffe W.F., Grennet M.H., Brewer R.A., Gamble K.L., Young M.E. (2013). Metabolic Dyssynchrony in Mice. Int. J. Obes..

[17] Bray M., Tsai J., Villegas-Montoya C., Boland B., Zackary B., Egbejimi O., Kueht M., Young M.E. (2010). Time-of-Day-Dependent Dietary Fat Consumption Influences Multiple Cardiometabolic Syndrome Parameters in Mice. Int. J. Obes..

[18] Vollmers C., Gill S., DiTacchio L., Pulivarthy S.R., Le H.D., Panda S. (2009). Time of feeding and the intrinsic circadian clock drive rhythms in hepatic gene expression. Proc. Natl. Acad. Sci. USA.

[19] Hatori M., Vollmers C., Zarrinpar A., DiTacchio L., Bushong E., Gill S., Leblanc M., Chaix A., Joens M., James A.J. (2012). Time restricted feeding without reducing caloric intake prevents metabolic diseases in mice fed a high fat diet. Cell Metab..

[20] Winget C., DeRoshia C., Markley C., Holley D. (1984). A review of human physiological and performance changes associated with desynchronosis of biological rhythms. Aviat. Space Environ. Med..

[21] Burch J., Yost M., Johnson W., Allen E. (2005). Melatonin, sleep, and shift work adaptation. J. Occup. Environ. Med..

[22] Gerstner J.R., Lyons L.C., Wright K.P., Loh D.H., Rawashdeh O., Eckel-Mahan K.L., Roman G.W. (2009). Cycling behavior and memory formation. J. Neurosci..

[23] Harrison E.M., Gorman M.R. (2012). Changing the waveform of circadian rhythms: Considerations for shift-work. Front. Neurol..

[24] Zaldívar M. (2013). Estadísticas de Accidentabilidad.

[25] Panda S. (2019). The arrival of circadian medicine. Nat. Rev. Endocrinol..

[26] Schrödinger E. (1944). What is Life? The Physical Aspect of the Living Cell.

[27] Krebs H. (1981). Reminiscences and Reflections.

[28] Semenov S.N., Kraft L.J., Ainla A., Zhao M., Baghbanzadeh M., Campbell V.E., Kang K., Fox J.M., Whitesides G.M. (2016). Autocatalytic, bistable, oscillatory networks of biologically relevant organic reactions. Nature.

[29] Hess B. (1979). The glycolytic oscillator. J. Exp. Biol..

[30] Rapp P. (1987). Why are so many biological systems periodic?. Prog. Neurobiol..

[31] Cedernaes J., Waldeck N., Bass J. (2019). Neurogenetic basis for circadian regulation of metabolism by the hypothalamus. Genes Dev..

[32] Hatanaka F., Matsubara C., Myung J., Yoritaka T., Kamimura N., Tsutsumi S., Kanai A., Suzuki Y., Sassone-Corsi P., Aburatani H. (2010). Genome-Wide Profiling of the Core Clock Protein BMAL1 Targets Reveals a Strict Relationship with Metabolism. Mol. Cell. Biol..

[33] Archer S., Viola A., Kyriakopoulou V., Von Schantz M., Dijk D. (2008). Inter-Individual Differences in Habitual Sleep Timing and Entrained Phase of Endogenous Circadian Rhythms of BMAL1, PER2 and PER3 mRNA in Human Leukocytes. Sleep.

[34] Zheng X., Sehgal A. (2008). Probing the relative importance of molecular oscillations in the circadian clock. Genetics.

[35] Rutkowski D.T., Hegde R.S. (2010). Regulation of basal cellular physiology by the homeostatic unfolded protein response. J. Cell Biol..

[36] Golombek D.A., Rosenstein R.E. (2010). Physiology of circadian entrainment. Physiol. Rev..

[37] Spanagel R., Pendyala G., Abarca C., Zghoul T., Sanchis-Segura C., Magnone M.C., Lascorz J., Depner M., Holzberg D., Soyka M. (2005). The clock gene Per2 influences the glutamatergic system and modulates alcohol consumption. Nat. Med..

[38] Dunlap J.C., Loros J.J. (2017). Making Time: Conservation of Biological Clocks from Fungi to Animals. Microbiol. Spectr..

[39] Rijo-Ferreira F., Carvalho T., Afonso C., Sanches-Vaz M., Costa R.M., Figueiredo L.M., Takahashi J.S. (2018). Sleeping sickness is a circadian disorder. Nat. Commun..

[40] Henslee E.A., Crosby P., Kitcatt S.J., Parry J.S.W., Bernardini A., Abdallat R.G., Braun G., Fatoyinbo H.O., Harrison E.J., Edgar R.S. (2017). Rhythmic potassium transport regulates the circadian clock in human red blood cells. Nat. Commun..

[41] Bass J.T. (2017). The circadian clock system’s influence in health and disease. Genome Med..

[42] Klevecz R.R., Li C.M., Marcus I., Frankel P.H. (2008). Collective behavior in gene regulation: The cell is an oscillator, the cell cycle a developmental process. FEBS J..

[43] Zhang H., Wang L., Shi K., Shan D., Zhu Y., Wang C., Bai Y., Yan T., Zheng X., Kong J. (2019). Apple tree flowering is mediated by low level of melatonin under the regulation of seasonal light signal. J. Pineal Res..

[44] Pilorz V., Astiz M., Heinen K.O., Rawashdeh O., Oster H. (2019). The concept of coupling in the mammalian circadian clock-network. J. Mol. Biol..

[45] Taylor S.R., Doyle F.J., Petzold L.R. (2008). Oscillator model reduction preserving the phase response: Application to the circadian clock. Biophys. J..

[46] Weinert D. (2010). Circadian temperature variation and ageing. Ageing Res. Rev..

[47] Greco C.M., Sassone–Corsi P. (2019). Circadian blueprint of metabolic pathways in the brain. Nat. Rev. Neurosci..

[48] Brown L.A., Fisk A.S., Pothecary C.A., Peirson S.N. (2019). Telling the time with a broken clock: Quantifying circadian disruption in animal models. Biology.

[49] Hastings M.H., Reddy A.B., Maywood E.S. (2003). A clockwork web: Circadian timing in brain and periphery, in health and disease. Nat. Rev. Neurosci..

[50] Shellock F., Rubin S., Ellrodt A., Muchlinski A., Brown H., Swan H. (1983). Unusual core temperature decrease in exercising heart-failure patients. J. Appl. Physiol..

[51] Rubin S. (1987). Core temperature regulation of heart rate during exercise in humans. J. Appl. Physiol..

[52] Waterhouse J., Drust B., Weinert D., Edwards B., Gregson W., Atkinson G., Kao S., Aizawa S., Reilly T. (2005). The circadian rhythm of core temperature: Origin and some implications for exercise performance. Chronobiol. Int..

[53] Reilly T., Atkinson G., Edwards B., Waterhouse J., Farrelly K., Fairhurst E. (2007). Diurnal variation in temperature, mental and physical performance, and tasks specifically related to football (soccer). Chronobiol. Int..

[54] Shellock F.G., Riedinger M.S., Fishbein M.C., Shah P.K. (1985). Prevalence of brown adipose tissue in chronic congestive heart failure secondary to coronary heart disease. Am. J. Cardiol..

[55] Cannon B., Nedergaard J. (2004). Brown Adipose Tissue: Function and Physiological Significance. Physiol. Rev..

[56] Au-Yong I.T.H., Thorn N., Ganatra R., Perkins A.C., Symonds M.E. (2009). Brown adipose tissue and seasonal variation in humans. Diabetes.

[57] Carrier J., Paquet J., Morettini J., Touchette É. (2002). Phase advance of sleep and temperature circadian rhythms in the middle years of life in humans. Neurosci. Lett..

[58] Van Someren E. (2000). More than a marker: Interaction between the circadian regulation of temperature and sleep, age-related changes, and treatment possibilities. Chronobiol. Int..

[59] Zulley J., Wever R., Aschoff J. (1981). The dependence of onset and duration of sleep on th circadian rhythm of rectal temperature. Pflug. Arch..

[60] Brown S.A., Zumbrunn G., Fleury-Olela F., Preitner N., Schibler U. (2002). Rhythms of mammalian body temperature can sustain peripheral circadian clocks. Curr. Biol..

[61] Buhr E., Yoo S., Takahashi J. (2010). Temperature as a universal resetting cue for mammalian circadian oscillators. Science.

[62] Young M., Bray M. (2007). Potential Role for Peripheral Circadian Clock Dyssynchrony in the Pathogenesis of Cardiovascular Dysfunction. Sleep Med. Rev..

[63] Terazono H., Mutoh T., Yamaguchi S., Kobayashi M., Akiyama M., Udo R., Ohdo S., Okamura H., Shibata S. (2003). Adrenergic regulation of clock gene expression in mouse liver. Proc. Natl. Acad. Sci. USA.

[64] Serón-Ferré M., Torres C., Parraguez V.H., Vergara M., Valladares L., Forcelledo M.L., Constandil L., Valenzuela G.J. (2002). Perinatal neuroendocrine regulation. Development of the circadian time-keeping system. Mol. Cell. Endocrinol..

[65] Seron-Ferre M., Valenzuela G.J., Torres-Farfan C. (2007). Circadian clocks during embryonic and fetal development. Birth Defects Res. Part C.

[66] Buxton O.M., Lee C.W., L’Hermite-Balériaux M., Turek F.W., Van Cauter E. (2003). Exercise elicits phase shifts and acute alterations of melatonin that vary with circadian phase. Am. J. Physiol..

[67] Mallis M., DeRoshia C. (2005). Circadian rhythms, sleep, and performance in space. Aviat. Space Environ. Med..

[68] Perreau-Lenz S., Pévet P., Buijs R., Kalsbeek A. (2004). The biological clock: The bodyguard of temporal homeostasis. Chronobiol. Int..

[69] Vandewalle G., Gais S., Schabus M., Balteau E., Carrier J., Darsaud A., Sterpenich V., Albouy G., Dijk D.J., Maquet P. (2007). Wavelength-dependent modulation of brain responses to a working memory task by daytime light exposure. Cereb. Cortex.

[70] Mattson M.P. (2008). Hormesis defined. Ageing Res. Rev..

[71] Liu A., Welsh D., Ko C., Tran H., Zhang E., Priest A., Buhr E., Singer O., Meeker K., Verma I. (2007). Intercellular coupling confers robustness against mutations in the SCN circadian clock network. Cell.

[72] Liu C., Li S., Liu T., Borjigin J., Lin J. (2007). Transcriptional coactivator PGC-1alpha integrates the mammalian clock and energy metabolism. Nature.

[73] Gielen S., Schuler G., Adams V. (2010). Cardiovascular effects of exercise training: Molecular mechanisms. Circulation.

[74] Jilg A., Bechstein P., Saade A., Dick M., Li T.X., Tosini G., Rami A., Zemmar A., Stehle J.H. (2019). Melatonin modulates daytime-dependent synaptic plasticity and learning efficiency. J. Pineal Res..

[75] Piazza E.A., Hasenfratz L., Hasson U., Lew-Williams C. (2019). Infant and adult brains are coupled to the dynamics of natural communication. bioRxiv.

[76] Gwinner E. (1996). Circadian and circannual programmes in avian migration. J. Exp. Biol..

[77] Dawson D., Gibbon S., Singh P. (1996). The hypothermic effect of melatonin on core body temperature: Is more better?. J. Pineal Res..

[78] Cermakian N., Sassone-Corsi P. (2000). Multilevel regulation of the circadian clock. Nat. Rev. Mol. Cell Biol..

[79] Reppert S., Weaver D. (2001). Molecular analysis of mammalian circadian rhythms. Annu. Rev. Physiol..

[80] Richter H., Torres-Farfán C., Rojas-García P., Campino C., Torrealba F., Serón-Ferré M. (2004). The circadian timing system: Making sense of day/night gene expression. Biol. Res..

[81] Welsh D.K., Takahashi J.S., Kay S.A. (2010). Suprachiasmatic Nucleus: Cell Autonomy and Network Properties. Annu. Rev. Physiol..

[82] Erren T., Reiter R. (2015). Melatonin: A universal time messenger. Neuroendocrinol. Lett..

[83] Manchester L.C., Coto-Montes A., Boga J.A., Andersen L.P.H., Zhou Z., Galano A., Vriend J., Tan D.X., Reiter R.J. (2015). Melatonin: An ancient molecule that makes oxygen metabolically tolerable. J. Pineal Res..

[84] Reiter R.J., Rosales-Corral S., Tan D.X., Jou M.J., Galano A., Xu B. (2017). Melatonin as a mitochondria-targeted antioxidant: One of evolution’s best ideas. Cell. Mol. Life Sci..

[85] Roopin M., Yacobi Y.Z., Levy O. (2013). Occurrence, diel patterns, and the influence of melatonin on the photosynthetic performance of cultured HA-related Symbiodinium. J. Pineal Res..

[86] Dodd A., Salathia N., Hall A., Kévei E., Tóth R., Nagy F., Hibberd J., Millar A., Webb A. (2005). Plant Circadian Clocks Increase Photosynthesis, Growth, Survival, and Competitive Advantage. Science.

[87] Andrés-Colás N., Perea-García A., Puig S., Peñarrubia L. (2010). Deregulated copper transport affects Arabidopsis development especially in the absence of environmental cycles. Plant Physiol..

[88] Mazars C., Thuleau P., Lamotte O., Bourque S. (2010). Cross-talk between ROS and calcium in regulation of nuclear activities. Mol. Plant.

[89] Tan X., Long W., Zeng L., Ding X., Cheng Y., Zhang X., Zou X. (2019). Melatonin-induced transcriptome variation of rapeseed seedlings under salt stress. Int. J. Mol. Sci..

[90] Suofu Y., Li W., Jean-Alphonse F.G., Jia J., Khattar N.K., Li J., Baranov S.V., Leronni D., Mihalik A.C., He Y. (2017). Dual role of mitochondria in producing melatonin and driving GPCR signaling to block cytochrome c release. Proc. Natl. Acad. Sci. USA.

[91] Hardeland R. (2018). Melatonin and inflammation—Story of a double-edged blade. J. Pineal Res..

[92] Hardeland R. (2019). Aging, melatonin, and the pro-and anti-inflammatory networks. Int. J. Mol. Sci..

[93] Blask D.E. (2009). Melatonin, sleep disturbance and cancer risk. Sleep Med. Rev..

[94] Alvarez-Artime A., Cernuda-Cernuda R., Cepas V., Gonzalez-Menendez P., Fernadez-Vega S., Quiros-Gonzalez I., Sainz R.M., Mayo J.C. (2020). Melatonin-Induced Cytoskeleton Reorganization Leads to Inhibition of Melanoma Cancer Cell Proliferation. Int. J. Mol. Sci..

[95] Chen Y., Tjong Y.W., Ip S.F., Tipoe G.L., Fung M.L. (2005). Melatonin enhances the hypoxic response of rat carotid body chemoreceptor. J. Pineal Res..

[96] Gubin D., Gubin G., Waterhouse J., Weinert D. (2006). The circadian body temperature rhythm in the elderly: Effect of single daily melatonin dosing. Chronobiol. Int..

[97] Erden S. (2019). Hypothermia Associated with Melatonin Ingestion in a Child with Autism. Clin. Neuropharmacol..

[98] Weissová K., Škrabalová J., Skálová K., Bendová Z., Kopřivová J. (2019). The Effect of a Common Daily Schedule on Human Circadian Rhythms during the Polar Day in Svalbard: A Field Study. J. Circadian Rhythm..

[99] Kirsz K., Szczesna M., Molik E., Zieba D.A. (2017). Effects of ghrelin on nocturnal melatonin secretion in sheep: An in vitro and in vivo approach. J. Anim. Sci..

[100] Kirsz K., Szczęsna M., Biernat W., Molik E., Zięba D.A. (2020). Involvement of orexin A in nocturnal melatonin secretion into the cerebrospinal fluid and the blood plasma in seasonal sheep. Gen. Comp. Endocrinol..

[101] Pendergast J.S., Branecky K.L., Huang R., Niswender K.D., Yamazaki S. (2014). Wheel-running activity modulates circadian organization and the daily rhythm of eating behavior. Front. Psychol..

[102] Adamovich Y., Ladeuix B., Golik M., Koeners M.P., Asher G. (2017). Rhythmic Oxygen Levels Reset Circadian Clocks through HIF1α. Cell Metab..

[103] Figueiro M. (2008). A proposed 24 h lighting scheme for older adults. Light. Res. Technol..

[104] Figueiro M.G., Bierman A., Rea M.S. (2008). Retinal mechanisms determine the subadditive response to polychromatic light by the human circadian system. Neurosci. Lett..

[105] Cajochen C., Jud C., Münch M., Kobialka S., Wirz-Justice A., Albrecht U. (2006). Evening exposure to blue light stimulates the expression of the clock gene PER2 in humans. Eur. J. Neurosci..

[106] Touitou Y., Motohashi Y., Reinberg A., Touitou C., Bourdeleau P., Bogdan A., Auzéby A. (1990). Effect of shift work on the night-time secretory patterns of melatonin, prolactin, cortisol and testosterone. Eur. J. Appl. Physiol. Occup. Physiol..

[107] Frank A. (2000). Injuries related to shiftwork. Am. J. Prev. Med..

[108] Knutsson A. (2003). Health disorders of shift workers. Occup. Med..

[109] Taheri S., Lin L., Austin D., Young T., Mignot E. (2004). Short sleep duration is associated with reduced leptin, elevated ghrelin, and increased body mass index. PLoS Med..

[110] James F.O., Cermakian N., Boivin D.B. (2007). Circadian rhythms of melatonin, cortisol, and clock gene expression during simulated night shift work. Sleep.

[111] Kempenaers B., Borgström P., Loës P., Schlicht E., Valcu M. (2010). Artificial night lighting affects dawn song, extra-pair siring success, and lay date in songbirds. Curr. Biol..

[112] Do M.T.H. (2019). Melanopsin and the Intrinsically Photosensitive Retinal Ganglion Cells: Biophysics to Behavior. Neuron.

[113] Hankins M.W., Peirson S.N., Foster R.G. (2008). Melanopsin: An exciting photopigment. Trends Neurosci..

[114] Mure L., Rieux C., Hattar S., Cooper H. (2007). Melanopsin-dependent nonvisual responses: Evidence for photopigment bistability in vivo. J. Biol. Rhythm..

[115] Russell B. (1988). A History of Western Philosophy.

[116] Damjanovic A., Milovanovic S.D., Trajanovic N.N. (2015). Descartes and His Peculiar Sleep Pattern. J. Hist. Neurosci..

[117] Friedman M. (2018). Analysis, Nutrition, and Health Benefits of Tryptophan. Int. J. Tryptophan Res..

[118] Brainard G.C., Sliney D., Hanifin J.P., Glickman G., Byrne B., Greeson J.M., Jasser S., Gerner E., Rollag M.D. (2008). Sensitivity of the human circadian system to short-wavelength (420-nm) light. J. Biol. Rhythm..

[119] Kayumov L., Casper R.F., Hawa R.J., Perelman B., Chung S.A., Sokalsky S., Shapiro C.M. (2005). Blocking low-wavelength light prevents nocturnal melatonin suppression with no adverse effect on performance during simulated shift work. J. Clin. Endocrinol. Metab..

[120] Kayumov L., Lowe A., Rahman S., Casper R., Shapiro C. (2007). Prevention of melatonin suppression by nocturnal lighting: Relevance to cancer. Eur. J. Cancer Prev..

[121] Barger L., Lockley S., Rajaratnam S., Landrigan C. (2009). Neurobehavioral, health, and safety consequences associated with shift work in safety-sensitive professions. Curr. Neurol. Neurosci. Rep..

[122] Canazei M., Pohl W., Bliem H.R., Weiss E.M. (2017). Acute effects of different light spectra on simulated night-shift work without circadian alignment. Chronobiol. Int..

[123] Horowitz T.S., Cade B.E., Wolfe J.M., Czeisler C.A. (2001). Efficacy of bright light and sleep/darkness scheduling in alleviating circadian maladaptation to night work. Am. J. Physiol..

[124] Arendt J. (2006). Melatonin and human rhythms. Chronobiol. Int..

[125] Tapia M., Wulff-Zottele C., De Gregorio N., Lang M., Varela H., Serón-Ferré M.J., Vivaldi E.A., Araneda O.F., Silva-Urra J., Gunga H.C. (2018). Melatonin relations with respiratory Quotient Weaken on acute exposure to high altitude. Front. Physiol..

[126] Richalet J., Rutgers V., Bouchet P., Rymer J., Kéromès A., Duval-Arnould G., Rathat C. (1989). Diurnal variations of acute mountain sickness, colour vision, and plasma cortisol and ACTH at high altitude. Aviat. Space Environ. Med..

[127] Tekavcic-Pompe M., Tekavcic I. (2008). Color vision in the tritan axis is predominantly affected at high altitude. High Alt. Med. Biol..

[128] Willmann G., Ivanov I.V., Fischer M.D., Lahiri S., Pokharel R.K., Werner A., Khurana T.S. (2010). Effects on colour discrimination during long term exposure to high altitudes on Mt Everest. Br. J. Ophthalmol..

[129] Davies A.J., Morris D.S., Kalson N.S., Wright A.D., Imray C.H.E., Hogg C.R. (2011). Changes to colour vision on exposure to high altitude. J. R. Army Med. Corps.

[130] Tekavcic B., Milić R., Pompe M. (2017). Does Physical Fatigue Affect Color Vision?. Sport Med. Int. Open.

[131] Kaur C., Srinivasan K.N., Singh J., Peng C.M., Ling E.A. (2002). Plasma melatonin, pinealocyte morphology, and surface receptors/antigen expression on macrophages/microglia in the pineal gland following a high-altitude exposure. J. Neurosci. Res..

[132] Frisch H., Waldhauser F., Waldhör T., Müllner-Eidenböck A., Neupane P., Schweitzer K. (2004). Increase in 6-hydroxymelatonin excretion in humans during ascent to high altitudes. J. Clin. Endocrinol. Metab..

[133] Klemm P., Hurst J., Dias Blak M., Herrmann T., Melchinger M., Bartz-Schmidt K.U., Zeck G., Schultheiss M., Spitzer M.S., Schnichels S. (2019). Hypothermia protects retinal ganglion cells against hypoxia-induced cell death in a retina organ culture model. Clin. Exp. Ophthalmol..

[134] Pires-Lapa M.A., Carvalho-Sousa C.E., Cecon E., Fernandes P.A., Markus R.P. (2018). β-Adrenoceptors trigger melatonin synthesis in phagocytes. Int. J. Mol. Sci..

[135] Utrillas M.P., Marín M.J., Esteve A.R., Salazar G., Suarez H., Castillo J., Martínez-Lozano J.A. (2016). UVER and UV index at high altitude in Northwestern Argentina. J. Photochem. Photobiol. B.

[136] Yasukouchi A., Maeda T., Hara K., Furuune H. (2019). Non-visual effects of diurnal exposure to an artificial skylight, including nocturnal melatonin suppression. J. Physiol. Anthropol..

[137] Moldavan M., Allen C. (2010). Retinohypothalamic tract synapses in the rat suprachiasmatic nucleus demonstrate short-term synaptic plasticity. J. Neurophysiol..

[138] Zucker R.S., Regehr W.G. (2002). Short-Term Synaptic Plasticity. Annu. Rev. Physiol..

[139] Himadri P., Kumari S.S., Chitharanjan M., Dhananjay S. (2010). Role of oxidative stress and inflammation in hypoxia-induced cerebral edema: A molecular approach. High Alt. Med. Biol..

[140] Reiter R.J., Tan D.X., Rosales-Corral S., Galano A., Zhou X.J., Xu B. (2018). Mitochondria: Central organelles for melatonins antioxidant and anti-aging actions. Molecules.

[141] Jung-Hynes B., Ahmad N. (2009). SIRT1 controls circadian clock circuitry and promotes cell survival: A connection with age-related neoplasms. FASEB J..

[142] Vitaterna M.H., Ko C.H., Chang A.M., Buhr E.D., Fruechte E.M., Schook A., Antoch M.P., Turek F.W., Takahashi J.S. (2006). The mouse Clock mutation reduces circadian pacemaker amplitude and enhances efficacy of resetting stimuli and phase-response curve amplitude. Proc. Natl. Acad. Sci. USA.

[143] Hablitz L.M., Molzof H.E., Abrahamsson X.E., Cooper J.M., Prosser R.A., Gamble X.L. (2015). GIRK channels mediate the nonphotic effects of exogenous melatonin. J. Neurosci..

[144] Passarella S., Duong M. (2008). Diagnosis and treatment of insomnia. Am. J. Health Syst. Pharm..

[145] Bonnefond A., Froguel P. (2017). Disentangling the Role of Melatonin and its Receptor MTNR1B in Type 2 Diabetes: Still a Long Way to Go?. Curr. Diabetes Rep..

[146] Kaur H., Mukherjee S., Baluska F., Bhatla S.C. (2015). Regulatory roles of serotonin and melatonin in abiotic stress tolerance in plants. Plant Signal. Behav..

[147] Guo Z., Jin H., Sun H., Zhao Y., Liu J., Ma H., Sun X., Yang Y. (2018). Antioxidant Melatonin: Potential Functions in Improving Cerebral Autoregulation After Subarachnoid Hemorrhage. Front. Physiol..

[148] Ohashi N., Ishigaki S., Isobe S. (2019). The pivotal role of melatonin in ameliorating chronic kidney disease by suppression of the renin–angiotensin system in the kidney. Hypertens. Res..

[149] Nishi E., Almeida V., Amaral F., Simon K., Futuro-Neto H., Pontes R., Cespedes J., Campos R., Bergamaschi C. (2019). Melatonin attenuates renal sympathetic overactivity and reactive oxygen species in the brain in neurogenic hypertension. Hypertens. Res..

[150] Reiter R.J., Tan D.X., Manchester L.C., Qi W. (2001). Biochemical reactivity of melatonin with reactive oxygen and nitrogen species: A review of the evidence. Cell Biochem. Biophys..

[151] Vriend J., Reiter R.J. (2016). Melatonin and the von Hippel-Lindau/HIF-1 oxygen sensing mechanism: A review. Biochim. Biophys. Acta.

[152] Blanco S., Hernández R., Franchelli G., Ramos-Álvarez M.M., Peinado M.Á. (2017). Melatonin influences NO/NOS pathway and reduces oxidative and nitrosative stress in a model of hypoxic-ischemic brain damage. Nitric Oxide.

[153] Arnao M.B., Hernández-Ruiz J. (2014). Melatonin: Plant growth regulator and/or biostimulator during stress?. Trends Plant Sci..

[154] Zhang H.J., Zhang N., Yang R.C., Wang L., Sun Q.Q., Li D.B., Cao Y.Y., Weeda S., Zhao B., Ren S. (2014). Melatonin promotes seed germination under high salinity by regulating antioxidant systems, ABA and GA4 interaction in cucumber (*Cucumis sativus* L.). J. Pineal Res..

[155] Cao Y.Y., Qi C.D., Li S., Wang Z., Wang X., Wang J., Ren S., Li X., Zhang N., Guo Y.D. (2019). Melatonin Alleviates Copper Toxicity via Improving Copper Sequestration and ROS Scavenging in Cucumber. Plant Cell Physiol..

[156] Izon G., Zerkle A.L., Williford K.H., Farquhar J., Poulton S.W., Claire M.W. (2017). Biological regulation of atmospheric chemistry en route to planetary oxygenation. Proc. Natl. Acad. Sci. USA.

[157] Murray A.J., Horscroft J.A. (2016). Mitochondrial function at extreme high altitude. J. Physiol..

[158] Jefferson J.A., Simoni J., Escudero E., Hurtado M.E., Swenson E.R., Wesson D.E., Schreiner G.F., Schoene R.B., Johnson R.J., Hurtado A. (2004). Increased oxidative stress following acute and chronic high altitude exposure. High Alt. Med. Biol..

[159] Behn C., Araneda O.F., Llanos A.J., Celedón G., González G. (2007). Hypoxia-related lipid peroxidation: Evidences, implications and approaches. Respir. Physiol. Neurobiol..

[160] Cable N.T., Drust B., Gregson W.A. (2007). The impact of altered climatic conditions and altitude on circadian physiology. Physiol. Behav..

[161] Mortola J.P. (2017). Gender and the circadian pattern of body temperature in normoxia and hypoxia. Respir. Physiol. Neurobiol..

[162] Pei J.F., Li X.K., Li W.Q., Gao Q., Zhang Y., Wang X.M., Fu J.Q., Cui S.S., Qu J.H., Zhao X. (2019). Diurnal oscillations of endogenous H_2_O_2_ sustained by p66Shc regulate circadian clocks. Nat. Cell Biol..

[163] Wu Y.Z., Zhang L., Wu Z.X., Shan T.T., Xiong C. (2019). Berberine Ameliorates Doxorubicin-Induced Cardiotoxicity via a SIRT1/p66Shc-Mediated Pathway. Oxid. Med. Cell. Longev..

[164] Hao C., Wu X., Zhou R., Zhang H., Zhou Y., Wang X., Feng Y., Mei L., He C., Cai X. (2019). Downregulation of p66Shc can reduce oxidative stress and apoptosis in oxidative stress model of marginal cells of stria vascularis in Sprague Dawley rats. Drug Des. Devel. Ther..

[165] Del Olmo M., Kramer A., Herzel H. (2019). A robust model for circadian redox oscillations. Int. J. Mol. Sci..

[166] Kim Y.K., Hammerling U. (2020). The mitochondrial PKCδ/retinol signal complex exerts real-time control on energy homeostasis. Biochim. Biophys. Acta.

[167] Kumar S. (2020). Physical activity pacifies the problematic p66Shc. Eur. J. Prev. Cardiol..

[168] Connett R.J., Honig C.R., Gayeski T.E.J., Brooks G.A. (1990). Defining hypoxia: A systems view of VO2, glycolysis, energetics, and intracellular PO2. J. Appl. Physiol..

[169] Ashkenazi I., Ribak J., Avgar D., Klepfish A. (1982). Altitude and hypoxia as phase shift inducers. Aviat. Space Environ. Med.

[170] Bishop B., Silva G., Krasney J., Salloum A., Roberts A., Nakano H., Shucard D., Rifkin D., Farkas G. (2000). Circadian rhythms of body temperature and activity levels during 63 h of hypoxia in the rat. Am. J. Physiol..

[171] Coste O., Beaumont M., Batéjat D., Van Beers P., Charbuy H., Touitou Y. (2004). Hypoxic depression of melatonin secretion after simulated long duration flights in man. J. Pineal Res..

[172] Mortola J.P. (2007). Correlations between the circadian patterns of body temperature, metabolism and breathing in rats. Respir. Physiol. Neurobiol..

[173] Vanlalhriatpuia K., Chhakchhuak V., Moses S.K., Iyyer S.B., Kasture M.S., Shivagaje A.J., Rajneesh B.J., Joshi D.S. (2007). Effects of altitude on circadian rhythm of adult locomotor activity in Himalayan strains of Drosophila helvetica. J. Circadian Rhythm..

[174] Saiki C., Mortola J. (1995). Hypoxia abolishes the morning-night differences of metabolism and ventilation in 6-day-old rats. Can. J. Physiol. Pharmacol..

[175] Mortola J.P., Seifert E.L. (2000). Hypoxic depression of circadian rhythms in adult rats. J. Appl. Physiol..

[176] Vargas M., Jiménez D., León-Velarde F., Osorio J., Mortola J. (2001). Circadian patterns in men acclimatized to intermittent hypoxia. Respir. Physiol..

[177] Bosco G., Ionadi A., Panico S., Faralli F., Gagliardi R., Data P., Mortola J. (2003). Effects of hypoxia on the circadian patterns in men. High Alt. Med. Biol..

[178] Chilov D., Hofer T., Bauer C., Wenger R.H., Gassmann M. (2001). Hypoxia affects expression of circadian genes PER1 and CLOCK in mouse brain. FASEB J..

[179] Kwarecki K., Krawczyk J. (1989). Comparison of the circadian rhythm in cell proliferation in corneal epithelium of male rats studied under normal and hypobaric (hypoxic) conditions. Chronobiol. Int..

[180] Joseph V., Mamet J., Lee F., Dalmaz Y., Van Reeth O. (2002). Prenatal hypoxia impairs circadian synchronisation and response of the biological clock to light in adult rats. J. Physiol..

[181] Mortola J.P., Lanthier C. (2004). Scaling the amplitudes of the circadian pattern of resting oxygen consumption, body temperature and heart rate in mammals. Comp. Biochem. Physiol..

[182] Piccione G., Caola G., Mortola J.P. (2005). Scaling the daily oscillations of breathing frequency and skin temperature in mammals. Comp. Biochem. Physiol..

[183] De Goot H., Littauer A. (1989). Hypoxia, reactive oxygen, and cell injury. Free Radic. Biol. Med..

[184] Araneda O.F., García C., Lagos N., Quiroga G., Cajigal J., Salazar M.P., Behn C. (2005). Lung oxidative stress as related to exercise and altitude. Lipid peroxidation evidence in exhaled breath condensate: A possible predictor of acute mountain sickness. Eur. J. Appl. Physiol..

[185] Celedón G., González G., Sotomayor C.P., Behn C. (1998). Membrane lipid diffusion and band 3 protein changes in human erythrocytes due to acute hypobaric hypoxia. Am. J. Physiol..

[186] González G., Celedón G., Sandoval M., González G.E., Ferrer V., Astete R., Behn C. (2002). Hypobaric hypoxia-reoxygenation diminishes band 3 protein functions in human erythrocytes. Pflug. Arch. Eur. J. Physiol..

[187] Hartman P., Ponder R., Lo H.H., Ishii N. (2004). Mitochondrial oxidative stress can lead to nuclear hypermutability. Mech. Ageing Dev..

[188] Bailey D.M., Davies B., Young I.S. (2001). Intermittent hypoxic training: Implications for lipid peroxidation induced by acute normoxic exercise in active men. Clin. Sci..

[189] Richalet J.P., Hornych A., Rathat C., Aumont J., Larmignat P., Rémy P. (1991). Plasma prostaglandins, leukotrienes and thromboxane in acute high altitude hypoxia. Respir. Physiol..

[190] Gonzalez-Candia A., Veliz M., Carrasco-Pozo C., Castillo R.L., Cárdenas J.C., Ebensperger G., Reyes R.V., Llanos A.J., Herrera E.A. (2019). Antenatal melatonin modulates an enhanced antioxidant/pro-oxidant ratio in pulmonary hypertensive newborn sheep. Redox Biol..

[191] Sagoo R.S., Hutchinson C.E., Wright A., Handford C., Parsons H., Sherwood V., Wayte S., Nagaraja S., Ng’Andwe E., Wilson M.H. (2017). Magnetic Resonance investigation into the mechanisms involved in the development of high-altitude cerebral edema. J. Cereb. Blood Flow Metab..

[192] Fan C., Zhao Y., Yu Q., Yin W., Liu H., Lin J., Yang T., Fan M., Gesang L., Zhang J. (2016). Reversible Brain Abnormalities in People Without Signs of Mountain Sickness during High-Altitude Exposure. Sci. Rep..

[193] Dwarakanath R.S., Sahar S., Reddy M.A., Castanotto D., Rossi J.J., Natarajan R. (2004). Regulation of monocyte chemoattractant protein-1 by the oxidized lipid, 13-hydroperoxyoctadecadienoic acid, in vascular smooth muscle cells via nuclear factor-kappa B (NF-κB). J. Mol. Cell. Cardiol..

[194] Cummins E.P., Berra E., Comerford K.M., Ginouves A., Fitzgerald K.T., Seeballuck F., Godson C., Nielsen J.E., Moynagh P., Pouyssegur J. (2006). Prolyl hydroxylase-1 negatively regulates IκB kinase-β, giving insight into hypoxia-induced NFκB activity. Proc. Natl. Acad. Sci. USA.

[195] Napetschnig J., Wu H. (2013). Molecular Basis of NF-κB Signaling. Annu. Rev. Biophys..

[196] Ryan S., Taylor C.T., McNicholas W.T. (2005). Selective activation of inflammatory pathways by intermittent hypoxia in obstructive sleep apnea syndrome. Circulation.

[197] Ryan S., Taylor C.T., McNicholas W.T. (2006). Predictors of elevated nuclear factor-κB-dependent genes in obstructive sleep apnea syndrome. Am. J. Respir. Crit. Care Med..

[198] Greenberg H., Ye X., Wilson D., Htoo A.K., Hendersen T., Liu S.F. (2006). Chronic intermittent hypoxia activates nuclear factor-κB in cardiovascular tissues in vivo. Biochem. Biophys. Res. Commun..

[199] Ham M., Kaunitz J.D. (2007). Gastroduodenal defense. Curr. Opin. Gastroenterol..

[200] Fruehauf H., Vavricka S.R., Lutz T.A., Gassmann M., Wojtal K.A., Erb A., Maggiorini M., Schwizer W., Fried M., Fox M. (2019). Evaluation of Acute Mountain Sickness by Unsedated Transnasal Esophagogastroduodenoscopy at High Altitude. Clin. Gastroenterol. Hepatol..

[201] Cheng J., Yang H.L., Gu C.J., Liu Y.K., Shao J., Zhu R., He Y.Y., Zhu X.Y., Li M.Q. (2019). Melatonin restricts the viability and angiogenesis of vascular endothelial cells by suppressing HIF-1α/ROS/VEGF. Int. J. Mol. Med..

[202] Paick S., Choi W.S. (2019). Varicocele and Testicular Pain: A Review. World J. Mens. Health.

[203] Zhu S.M., Rao T., Yang X., Ning J.Z., Yu W.M., Ruan Y., Yuan R., Li C.L., Jiang K., Hu W. (2017). Autophagy may play an important role in varicocele. Mol. Med. Rep..

[204] Goren M.R., Kilinc F., Kayaselcuk F., Ozer C., Oguzulgen I., Hasirci E. (2017). Effects of experimental left varicocele repair on hypoxia-inducible factor-1α and vascular endothelial growth factor expressions and angiogenesis in rat testis. Andrologia.

[205] Ramsey K.M., Bass J. (2009). Obeying the clock yields benefits for metabolism. Proc. Natl. Acad. Sci. USA.

[206] McClung C. (2007). Circadian Genes, Rhythms and the Biology of Mood Disorders. Pharmacol. Ther..

[207] Roybal K., Theobold D., Graham A., DiNieri J.A., Russo S.J., Krishnan V., Chakravarty S., Peevey J., Oehrlein N., Birnbaum S. (2007). Mania-like behavior induced by disruption of CLOCK. Proc. Natl. Acad. Sci. USA.

[208] Partonen T., Treutlein J., Alpman A., Frank J., Johansson C., Depner M., Aron L., Rietschel M., Wellek S., Soronen P. (2007). Three circadian clock genes Per2, Arntl, and Npas2 contribute to winter depression. Ann. Med..

[209] McClung C.A., Sidiropoulou K., Vitaterna M., Takahashi J.S., White F.J., Cooper D.C., Nestler E.J. (2005). Regulation of dopaminergic transmission and cocaine reward by the Clock gene. Proc. Natl. Acad. Sci. USA.

[210] Kovanen L., Saarikoski S.T., Haukka J., Pirkola S., Aromaa A., Lönnqvist J., Partonen T. (2010). Circadian clock gene polymorphisms in alcohol use disorders and alcohol consumption. Alcohol. Alcohol..

[211] Xu Y., Padiath Q.S., Shapiro R.E., Jones C.R., Wu S.C., Saigoh N., Saigoh K., Ptáček L.J., Fu Y.H. (2005). Functional consequences of a CKIδ mutation causing familial advanced sleep phase syndrome. Nature.

[212] Haba-Rubio J. (2005). Psychiatric aspects of organic sleep disorders. Dialogues Clin. Neurosci..

[213] Brown G.M., Pandi-Perumal S.R., Trakht I., Cardinali D.P. (2009). Melatonin and its relevance to jet lag. Travel Med. Infect. Dis..

[214] Monk T.H., Buysse D.J., Carrier J., Kupfer D.J. (2000). Inducing jet-lag in older people: Directional asymmetry. J. Sleep Res..

[215] Sookoian S., Gemma C., Fernández Gianotti T., Burgueño A., Alvarez A., González C.D., Pirola C.J. (2007). Effects of rotating shift work on biomarkers of metabolic syndrome and inflammation. J. Intern. Med..

[216] Spiegel K., Leproult R., Van Cauter E. (1999). Impact of sleep debt on metabolic and endocrine function. Lancet.

[217] Patel S.R., Ayas N.T., Malhotra M.R., White D.P., Schernhammer E.S., Speizer F.E., Stampler M.J., Hu F.B. (2004). A prospective study of sleep duration and mortality risk in women. Sleep.

[218] Davidson A.J., Sellix M.T. (2006). Chronic jet- lag increases mortality in aged mice. Curr. Biol..

[219] Gómez-Abellán P., Hernández-Morante J., Luján J., Madrid J., Garaulet M. (2008). Clock genes are implicated in the human metabolic syndrome. Int. J. Obes..

[220] Takahashi J.S., Hong H.K., Ko C.H., McDearmon E.L. (2008). Implications for Physiology and Disease. Nat. Rev. Genet..

[221] Scheer F.A.J.L., Hilton M.F., Mantzoros C.S., Shea S.A. (2009). Adverse metabolic and cardiovascular consequences of circadian misalignment. Proc. Natl. Acad. Sci. USA.

[222] Spiegel K., Tasali E., Penev P., Van Cauter E. (2004). Brief communication: Sleep curtailment in healthy young men is associated with decreased leptin levels, elevated ghrelin levels, and increased hunger and appetite. Ann. Intern. Med..

[223] Turek F.W., Joshu C., Kohsaka A., Lin E., Ivanova G., McDearmon E., Laposky A., Losee-Olson S., Easton A., Jensen D.R. (2005). Obesity and metabolic syndrome in circadian Clock mutant nice. Science.

[224] Guzmán-Ruiz R., Somoza B., Gil-Ortega M., Merino B., Cano V., Attané C., Castan-Laurell I., Valet P., Fernández-Alfonso M.S., Ruiz-Gayo M. (2010). Sensitivity of cardiac carnitine palmitoyltransferase to malonyl-CoA is regulated by leptin: Similarities with a model of endogenous hyperleptinemia. Endocrinology.

[225] Pavanello S., Stendardo M., Mastrangelo G., Casillo V., Nardini M., Mutti A., Campisi M., Andreoli R., Boschetto P. (2019). Higher number of night shifts associates with good perception of work capacity and optimal lung function but correlates with increased oxidative damage and telomere attrition. BioMed Res. Int..

[226] Maury E., Ramsey K., Bass J. (2010). Circadian rhythms and metabolic syndrome: From experimental genetics to human disease. Circ. Res..

[227] Costa G. (2003). Shift work and occupational medicine: An overview. Occup. Med..

[228] Kroenke C.H., Spiegelman D., Manson J.A., Schernhammer E.S., Colditz G.A., Kawachi I. (2007). Work characteristics and incidence of type 2 diabetes in women. Am. J. Epidemiol..

[229] Coca A. (1994). Circadian rhythm and blood pressure control: Physiological and pathophysiological factors. J. Hypertens. Suppl..

[230] Goncharuk V., Van Heerikhuize J., Dai J., Swaab D., Buijs R. (2001). Neuropeptide changes in the suprachiasmatic nucleus in primary hypertension indicate functional impairment of the biological clock. J. Comp. Neurol..

[231] Gibson M., Williams W., Kriegsfeld L. (2009). Aging in the Circadian System: Considerations for Health, Disease Prevention, and Longevity. Exp. Gerontol..

[232] Filipski E., Li X.M., Lévi F. (2006). Disruption of circadian coordination and malignant growth. Cancer Causes Control.

[233] Stevens R.G. (2009). Light-at-night, circadian disruption and breast cancer: Assessment of existing evidence. Int. J. Epidemiol..

[234] Zhu Y., Stevens R., Hoffman A., FitzGerald L., Kwon E., Ostrander E., Davis S., Zheng T., Stanford J. (2009). Testing the circadian gene hypothesis in prostate cancer: A population-based case-control study. Cancer Res..

[235] Hoffman E., Zheng T., Yi C., Stevens R., Ba Y., Zhang Y., Leaderer D., Holford T., Hansen J., Zhu Y. (2010). The core circadian gene cryptochrome 2 influences breast cancer risk, possibly by mediating hormone signaling. Cancer Prev. Res..

[236] Davis S., Mirick D., Stevens R. (2001). Night Shift Work, Light at Night, and Risk of Breast Cancer. J. Natl. Cancer Inst..

[237] Hansen J. (2006). Risk of breast cancer after night- and shift work: Current evidence and ongoing studies in Denmark. Cancer Causes Control.

[238] Schernhammer E.S., Laden F., Speizer F.E., Willet W.C., Hunter D.J., Kawachi I., Fuchs C.S., Colditz G.A. (2003). Night-shift work and risk of colorectal cancer in the Nurses’ Health Study. J. Natl. Cancer Inst..

[239] Reppert S.M., Weaver D.R. (2002). Coordination of circadian clocks in mammals. Nature.

[240] Erren T.C., Reiter R.J. (2009). Defining chronodisruption. J. Pineal Res..

[241] Preuss F., Tang Y., Laposky A., Arble D., Keshavarzian A., Turek F. (2008). Adverse effects of chronic circadian desynchronization in animals in a “challenging” environment. Am. J. Physiol. Regul. Integr. Comp. Physiol..

[242] Dallman M., Akana S., Pecoraro N., Warne J., La Fleur S., Foster M. (2007). Glucocorticoids, the etiology of obesity and the metabolic syndrome. Curr. Alzheimer Res..

[243] Scheer F.A.J.L., Ter Horst G.J., Van Der Vliet J., Buijs R.M. (2001). Physiological and anatomic evidence for regulation of the heart by suprachiasmatic nucleus in rats. Am. J. Physiol..

[244] Zhao H., Yin Z., Xiang H., Liao Z., Wang Z. (2017). Preliminary study on alterations of altitude road traffic in China from 2006 to 2013. PLoS ONE.

[245] Knott M., Classen S., Krasniuk S., Tippett M., Alvarez L. (2020). Insufficient sleep and fitness to drive in shift workers: A systematic literature review. Accid. Anal. Prev..

[246] Paul S., Gangwar A., Bhargava K., Khurana P., Ahmad Y. (2018). Diagnosis and prophylaxis for high-altitude acclimatization: Adherence to molecular rationale to evade high-altitude illnesses. Life Sci..

[247] Higgins J.P., Tuttle T., Higgins J.A. (2010). Altitude and the heart: Is going high safe for your cardiac patient?. Am. Heart J..

[248] Behn C., Dinamarca G.A., De Gregorio N.F., Lips V., Vivaldi E.A., Soza D., Guerra M.A., Jiménez R.F., Lecannelier E.A., Varela H. (2014). Age-related arrhythmogenesis on ascent and descent: “Autonomic conflicts” on hypoxia/reoxygenation at high altitude?. High Alt. Med. Biol..

[249] Roche E., Romero-Alvira D. (1994). Role of oxygen free radicals in altitude-related disorders. Med. Hypotheses.

[250] Vearrier D., Greenberg M.I. (2011). Occupational health of miners at altitude: Adverse health effects, toxic exposures, pre-placement screening, acclimatization, and worker surveillance. Clin. Toxicol..

[251] Firth P.G., Zheng H., Windsor J.S., Sutherland A.I., Imray C.H., Moore G.W.K., Semple J.L., Roach R.C., Salisbury R.A. (2008). Christmas 2008: Sport: Mortality on Mount Everest, 1921-2006: Descriptive study. BMJ.

[252] Firth P., Zheng H., Windsor J., Sutherland A., Imray C., Moore G., Semple J., Roach R., Salisbury R. (2008). Mortality on Mount Everest, 1921-2006: Descriptive study. BMJ.

[253] Fornasiero A., Savoldelli A., Skafidas S., Stella F., Bortolan L., Boccia G., Zignoli A., Schena F., Mourot L., Pellegrini B. (2018). Delayed parasympathetic reactivation and sympathetic withdrawal following maximal cardiopulmonary exercise testing (CPET) in hypoxia. Eur. J. Appl. Physiol..

[254] Hamm W., Von Stülpnagel L., Klemm M., Baylacher M., Rizas K.D., Bauer A., Brunner S. (2018). Deceleration Capacity of Heart Rate after Acute Altitude Exposure. High Alt. Med. Biol..

[255] Gianfredi V., Albano L., Basnyat B., Ferrara P., Count W. (2020). Does age have an impact on acute mountain sickness? A systematic review. J. Travel Med..

[256] Kuo T.B.J., Li J.Y., Kuo H.K., Chern C.M., Yang C.C.H. (2016). Differential changes and interactions of autonomic functioning and sleep architecture before and after 50 years of age. Age.

[257] Ray C. (2003). Melatonin attenuates the sympathetic nerve responses to ortho-static stress in humans. J. Physiol..

[258] Pechanova O., Paulis L., Simko F. (2014). Peripheral and central effects of melatonin on blood pressure regulation. Int. J. Mol. Sci..

[259] Baker J., Kimpinski K. (2018). Role of melatonin in blood pressure regulation: An adjunct anti-hypertensive agent. Clin. Exp. Pharmacol. Physiol..

[260] Rauch S., Schenk K., Strapazzon G., Dal Cappello T., Gatterer H., Palma M., Erckert M., Oberhuber L., Bliemsrieder B., Brugger H. (2019). Suspension syndrome: A potentially fatal vagally mediated circulatory collapse—An experimental randomized crossover trial. Eur. J. Appl. Physiol..

[261] Reno C.M., Bayles J., Huang Y., Oxspring M., Hirahara A.M., Dosdall D.J., Fisher S.J. (2019). Severe hypoglycemia–induced fatal cardiac arrhythmias are mediated by the parasympathetic nervous system in rats. Diabetes.

[262] Nishiyama K., Yasue H., Moriyama Y., Tsunoda R., Ogawa H., Yoshimura M., Kugiyama K. (2001). Acute effects of melatonin administration on cardiovascular autonomic regulation in healthy men. Am. Heart J..

[263] Doolen S., Krause D.N., Dubocovich M.L., Duckles S.P. (1998). Melatonin mediates two distinct responses in vascular smooth muscle. Eur. J. Pharmacol..

[264] Masana M.I., Doolen S., Ersahin C., Al-Ghoul W.M., Duckles S.P., Dubocovich M.L., Krause D.N. (2002). MT2 melatonin receptors are present and functional in rat caudal artery. J. Pharmacol. Exp. Ther..

[265] Campos L.A., Pereira V.L., Muralikrishna A., Albarwani S., Brás S., Gouveia S. (2013). Mathematical biomarkers for the autonomic regulation of cardiovascular system. Front. Physiol..

[266] Imenshahidi M., Karimi G., Hosseinzadeh H. (2020). Effects of melatonin on cardiovascular risk factors and metabolic syndrome: A comprehensive review. Naunyn. Schmiedebergs. Arch. Pharmacol..

[267] Green E.A., Black B.K., Biaggioni I., Paranjape S.Y., Bagai K., Shibao C., Okoye M.C., Dupont W.D., Robertson D., Raj S.R. (2014). Melatonin reduces tachycardia in postural tachycardia syndrome: A randomized, crossover trial. Cardiovasc. Ther..

[268] Wang S., Zhai X., Li S., McCabe M.F., Wang X., Rong P. (2015). Transcutaneous vagus nerve stimulation induces tidal melatonin secretion and has an antidiabetic effect in Zucker fatty rats. PLoS ONE.

